# Treatment of classical Hodgkin lymphoma in young adults aged 18–30 years with a modified paediatric Hodgkin lymphoma protocol. Results of a multicentre phase II clinical trial (CRUK/08/012)

**DOI:** 10.1111/bjh.16296

**Published:** 2019-11-11

**Authors:** William Townsend, Sarah Leong, Peter Hoskin, Patricia Diez, Pip Patrick, David Linch, Wai‐Lup Wong, Irfan Kayani, Bal Sanghera, Andre Lopes, Stephen Daw, Graham Collins, Laura Clifton‐Hadley, Kirit Ardeshna

**Affiliations:** ^1^ Department of Haematology University College London Hospitals NHS Foundation trust London UK; ^2^ Cancer Research UK and University College London Cancer Trials Centre London UK; ^3^ Marie Curie Research Wing Mount Vernon Cancer Centre Northwood UK; ^4^ National Radiotherapy Trials Quality Assurance Group Mount Vernon Cancer Centre Northwood UK; ^5^ Paul Strickland Scanner Centre Mount Vernon Cancer Centre Northwood UK; ^6^ Department of Nuclear Medicine University College Hospitals NHS Foundation Trust London UK; ^7^ Department of Paediatric Oncology University College Hospitals NHS Foundation Trust London UK; ^8^ Oxford Cancer and Haematology Centre Churchill Hospital Oxford UK

**Keywords:** Hodgkin lymphoma, paediatric, neuropathy

## Abstract

This phase II trial was designed to determine the safety and efficacy of a modified paediatric risk‐stratified protocol in young adults (18–30 years) with classical Hodgkin Lymphoma. The primary end‐point was neurotoxicity rate. The incidence of grade 3 neurotoxicity was 11% (80% CI, 5–19%); a true rate of neuropathy of >15% cannot be excluded. Neuropathy and associated deterioration in quality of life was largely reversible. The overall response rate was 100% with 40% complete remission (CR) rate. Twelve months disease‐free survival (DFS) was 91%. We demonstrate that a risk‐stratified paediatric combined modality treatment approach can be delivered to young adults without significant irreversible neuropathy.

Although there have been no randomised comparisons, children with classical Hodgkin lymphoma (cHL) treated with paediatric protocols appear to have better outcomes than adults with equivalent stage disease treated with ABVD (doxorubicin, bleomycin, vinblastine and dacarbazine). It is not known whether this is a function of age, due to differences in the biology of the disease, or because paediatric regimens are more effective.

The current standard of care for adults with cHL is ABVD or EscalatedBEACOPP (bleomycin, etoposide, doxorubicin, cyclophosphamide, vincristine, procarbazine and prednisolone) with the number of cycles and addition of radiotherapy dependent on stage and positron‐emission tomography (PET)‐guided response assessment (Radford *et al.*, 2015; Johnson *et al.*, 2016; Borchmann *et al.*, 2017).

In the paediatric setting, combined modality treatment is used in a risk‐stratified approach with patients allocated to treatment groups (TGs) according to stage (Rühl *et al.*, 2001; Mauz‐Körholz *et al.*, 2010; Dörffel *et al.*, 2013). The chemotherapy regimens used in paediatric studies include OEPA (vincristine, etoposide, prednisolone and doxorubicin) and COPP (cyclophosphamide, vincristine, procarbazine and prednisolone) or variations of these regimens. The chemotherapy protocols, radiotherapy fields and doses have been refined in a series of trials detailed in the supplementary files (Mauz‐Körholz *et al.*, 2010; Dörffel *et al.*, 2013).

There are significant differences between paediatric regimens and ABVD. Most pertinent to this trial is the greater intensity and cumulative dose of vinca alkaloids in paediatric regimens with a maximum of 14 doses of vincristine (1.5 mg/m^2^) in the paediatric protocol compared to 12 doses of vinblastine (6 mg/m^2^) with six cycles of ABVD. Other differences are presented in the supplementary files.

Paediatric regimens have never previously been assessed prospectively in adult patients with cHL. The aim of this trial was to investigate whether a modified version of the paediatric protocol as used in the Gesellschaft für Pädiatrische Onkologie und Hämatologie (GPOH)‐HD95 trial could be delivered to young adults without inducing excessive neurotoxicity (Dörffel *et al*, 2013).

## Patients and methods

This phase II, non‐randomised, open label, multicentre trial (ClinicalTrials.gov: NCT00666484) was designed to determine the safety and efficacy of a modified, risk‐stratified, combined modality paediatric regimen in young adults aged 18–30 years with a diagnosis of cHL. Full inclusion and exclusion criteria are listed in the supplementary files. The primary outcome measure was neurotoxicity, secondary outcome measures included response rate, DFS and quality of life (QoL).

The trial was managed by the Cancer Research UK and University College London Cancer Trials Centre. The protocol was approved by the national research ethics committee. Informed consent was obtained from all patients and the trial was conducted in accordance with the Declaration of Helsinki.

**Figure 1:**
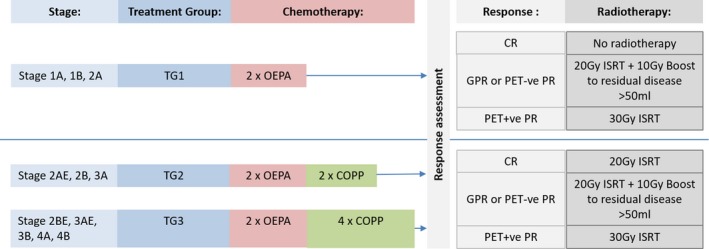
Trial schema.

Staging was performed according to the methods described in the supplementary data and patients were allocated into one of three treatment groups according to centrally reviewed staging scans (Fig 1).

Response was assessed 10–14 days after the last dose of chemotherapy according to the GPOH‐HD definitions in use at the time and the 2007 International Harmonisation Project Response criteria (Cheson *et al*, 2007), which predates and differs from the Lugano criteria currently used (Table SIV, and Data S1) (Mauz‐Körholz *et al.*, 2010; Cheson *et al*, 2014; Cheson *et al.*, 2016). All fluorodeoxyglucose (FDG)‐PET scans were performed in accredited centres according to a standardised protocol.

Toxicity was assessed according to the National Cancer Institute Common Terminology Criteria for Adverse Events version 3.0, radiotherapy toxicities were assessed according to Radiation Therapy Oncology Group criteria. The impact of neuropathy on QoL was assessed using the EORTC QLQ‐C30 QOL and Chemotherapy Induced Peripheral Neuropathy supplementary questionnaires (QLQ‐CIPN20).

Treatment in TG1 comprised two cycles of OEPA with no radiotherapy in patients who achieved CR. Treatment in TG2 comprised two cycles of OEPA and two cycles of COPP followed by radiotherapy. Patients in TG3 received two cycles of OEPA and four cycles of COPP followed by radiotherapy (Fig 1). See Data S1 for full dosing information.

Patients developing grade 3 peripheral neuropathy were switched to vinblastine (6 mg/m^2^) and vinca alkaloids were stopped if neurotoxicity further progressed.

Radiotherapy was commenced within four weeks of day 28 of the final cycle of chemotherapy for all patients except those in TG1 who achieved CR after chemotherapy. Radiotherapy volumes were determined by central review of baseline and end‐of‐chemotherapy scans;, full details of radiotherapy planning and delivery are provided in Data S1.

Statistical considerations and methods are provided in full in the supplementary data.

## Results

Fourty‐seven patients were recruited from eight UK centres between 2008 and 2011. The median follow‐up (censoring at death) was 3·3 years. One patient withdrew consent before starting chemotherapy, 46 patients received trial treatment, one patient was withdrawn on day 1 of cycle 1 due to a grade 3 reaction to etoposide (Figure S1).

The median age was 23 years (IQR 20–26); patient characteristics are presented in Table SI. The number of patients in TG1, TG2 and TG3 were 16 (36%), 11 (24%) and 18 (40%) respectively; the two patients who withdrew from the trial were not allocated a final TG.

With the exception of the two patients who withdrew from the trial, all patients received the full number of cycles of chemotherapy specified in the protocol. Treatment delivered is detailed in Table SII.

Of the 45 patients completing chemotherapy, four patients in TG1 achieved CR and accordingly did not receive radiotherapy. The remaining 41 patients received radiotherapy. Compliance with the radiotherapy protocol was centrally assessed prospectively in 23 cases with five minor and four major variations from protocol identified (Table SIV). All variations were corrected before patients proceeded to treatment.

The incidence of grade 3 neurotoxicity in the 46 patients who started treatment was 11% (two‐sided exact 80% CI, 5–19%). Five patients had one or more episodes of grade 3 neurotoxicity and there were no cases of grade 4 or 5 neurotoxicity (Table SVI). Severe neuropathy was not limited to patients in any particular TG, it occurred in three patients in TG2 and one patient in each of TGs 1 and 3; onset was during cycle 1 of OEPA in two patients, cycle 2 of OEPA in two patients and cycle 2 of COPP in one patient.

All cases of grade 3 neuropathy reverted to grade 0 with a median time to resolution of 91 days [interquartile range (IQR) 12–100 days]. In addition to the severe neuropathy, there were 32 episodes where the maximum recorded neurotoxicity was grade 1/2 in 19 patients (41%), this resolved to grade 0 in all except for one patient who reported persistent grade 2 neuropathy. The median time to resolution of grade 1/2 neuropathy was 74 days (IQR 18–240 days).

Haematological toxicities were the commonest severe toxicity with grade 3/4 neutropenia reported in 38 patients (83%) (Table SVII).

Three patients developed osteonecrosis of the hips or knees, grade 3/4 in two patients (4%) and grade 2 in one patient with grade 3 pain. The affected patients were 19, 20 and 29 years at trial entry and all were male. Osteonecrosis occurred in one patient in TG1 and two patients in TG3, the diagnosis was made >1 year after completion of chemotherapy in two of the patients. One patient required bilateral decompression of the femoral heads and hip replacement surgery.

Radiotherapy toxicities were reported in 56% of patients receiving radiotherapy (Table SVIII).

There was a significant deterioration in the sensory and motor neuropathy QoL scales between pretreatment and immediately after chemotherapy, mean difference in QoL 9·5 (99% CI 2·5–16·5, *P* < 0·001) and 10 (99% CI 2·9–17·0, *P* < 0·001) respectively, with no significant difference in autonomic neuropathy scale (*P* = 0·26). At 12 months after treatment, the sensory scale had improved and was no longer significantly worse than at baseline. The motor scale had improved but remained 2·6 points worse than pretreatment, which is not considered clinically important (99% CI 0·4–4·8, *P* = 0·003) (Figure S3).

The overall response rate (ORR) at end of chemotherapy was 100% (2‐sided exact 80% CI 95–100%) with 40% achieving CR according to local response assessment. The CR rates prior to radiotherapy in TG1, TG2 and TG3 were 25%, 55% and 44% respectively (Table 1). Male and female patients had similar CR rates of 41% and 39% respectively (*P* = 0·90). Central review of end‐of‐chemotherapy PET scans was performed for 39/45 patients who received at least one cycle of treatment and was negative in 28/39 patients reviewed (72%), corresponding to metabolic remission by International Harmonization Project (IHP) 2007 and Lugano 2014 criteria.

**Table 1:** Response to treatment (response at restaging after chemotherapy and before radiotherapy).

Response	All patients (*n* = 45)*			TG1 (*n* = 16)			TG2 (*n* = 11)			TG3 (*n* = 18)	
	*N*	%		*N*	%		*N*	%		*N*	%
Complete remission	18	40		4	25		6	55		8	44
Good partial remission	18	40		7	44		2	18		9	50
Partial remission	9	20		5	31		3	27		1	6
Overall response (CR + GPR + PR)	45	100		16	100		11	100		18	100
PET negative†	28	72		9	64		8	89		11	69

* Two patients withdrew from the trial, only 45 completed treatment.

^†^ PET response by central review: 39 patients had central review of PET at end of chemotherapy, central review was not completed in six patients (two in TG1, two in TG2 and two in TG3). Percentages are based on the total number of patients with available central review assessment.

Four patients have relapsed of whom one patient has died due to cHL; no patients achieving CR (*n* = 18) with a negative PET have relapsed. The 12‐month DFS rate is 91% (95% CI 78–97) (Figure S2).

## Discussion

In this trial we investigated whether delivering a paediatric‐style, risk‐stratified, combined modality regimen to young adults is feasible without inducing excessive neurotoxicity due to the intensive use of vinca alkaloids. We found that 11% of patients treated with this protocol developed grade 3 neuropathy, which was reversible in all cases, with a median time to resolution of 91 days. One patient with G1/2 neuropathy had persisting toxicity at last follow‐up. The upper limit of the 80% CI was higher than the predetermined unacceptable rate stated in the trial protocol of >15%; therefore we cannot exclude the possibility that the true rate of grade 3 neurotoxicity was >15%. Although an initial deterioration in neuropathy‐related QoL was recorded, this had improved 12 months after treatment. Overall, we conclude that the vinca alkaloid dosing used in this protocol in this age group is tolerable and associated neuropathy is reversible in most cases.

It is difficult to make direct comparisons with the neurotoxicity rate in other trials due to variations in reporting; however, it is similar to that reported in the paediatric GPOH‐HD‐2002 trial (Mauz‐Körholz *et al.*, 2010). Whilst it is higher than reported in adults after ABVD (Diehl, Franklin *et al.*, 2003), it may be comparable to the rate experienced after EscalatedBEACOPP, where 12·7% of adults treated with eight cycles reported grade 3/4 ‘nervous system’ adverse events (Engert *et al.*, 2012).

Of concern, three patients (6·5%) in this trial developed avascular necrosis. This is likely to be due to the high dose of corticosteroids in this regimen. It is known from studies of childhood acute lymphoblastic leukaemia (Mattano, Sather *et al.*, 2000) that avascular necrosis is more common in older children and with higher doses of steroids, which may indicate why we identified a high rate in this trial of young adults treated with high doses of corticosteroids. In a retrospective review of adult cHL patients treated in German Hodgkin Study Group trials, the cumulative incidence of osteonecrosis was 0·93% and was more frequent in male patients. Osteonecrosis was not specifically assessed in these studies and the true rate may be higher as indicated by a small series reporting a rate of 21% (Fosså *et al.*, 2011, Borchmann *et al.*, 2016). Osteonecrosis has not been specifically reported in the GPOH paediatric trials.

Whilst the sample size is too small to draw conclusions about the efficacy of this protocol in adults, the 100% response rate, high proportion of patients with a negative PET and high DFS in this trial are promising.

The use of radiotherapy in this trial reflects standard practice in paediatric trials at the time of protocol development. Subsequent work published in abstract form has demonstrated that radiotherapy can be omitted in children with a negative interim PET scan regardless of TG without impairing event‐free survival and much less radiotherapy is being used in current trials (Landman‐Parker *et al.*, 2016).

It is acknowledged that since this protocol was developed, standsardised PET reporting has been developed which differs from the PET scoring system used in this trial (Cheson *et al.*, 2016).

This trial demonstrates that a risk‐stratified approach using paediatric‐style treatment can be delivered to young adults aged 18–30 years without inducing unacceptable levels of severe irreversible peripheral neurotoxicity, and without impairing the ORR compared to historical cohorts. Results justify further testing of paediatric‐style treatment in adults and this trial has informed the design of an on‐going international trial using risk‐stratified treatment in children and young adults up to the age of 25 (EuroNet‐PHL‐C2 trial, NCT02684708).

## Supporting information


**Data S1**. Supplementary methods.
**Table SI**. Baseline patient characteristics.
**Table SII**. Number of patients who received less than 90% of the intended dose for each of the drugs in each cycle, by treatment group.
**Table SIII**. Schedule of investigations and follow‐up.
**Table SIV**. Radiotherapy outlining protocol variations (one major variation patient displayed two types of variation).
**Table SV**. FDG‐PET response categories.
**Table SVI**. Worst grade of neurotoxicity during the trial.
**Table SVII**. Non‐neuropathy toxicities: worst toxicity grade at any point in time of the trial (excluding neurotoxicity).
**Table SVIII**. Worst grade of radiotherapy toxicity reported six months after completing radiotherapy.
**Fig S1**. Consort diagram.
**Fig S2**. Disease‐free survival.
**Fig S3**. Sensory and motor quality‐of‐life scales. There was a significant deterioration in both scales between pretreatment and postchemotherapy. At 12 months post‐treatment, sensory scale was not significantly different to baseline but motor scale remained 2.6 points worse than at baseline.
