# Supplementary material for: Treatment of classical Hodgkin lymphoma in young adults aged 18–30 years with a modified paediatric Hodgkin lymphoma protocol. Results of a multicentre phase II clinical trial (CRUK/08/012)
Source: Br J Haematol. 2019 Nov 11;189(1):128–32. doi: 10.1111/bjh.16296 (PMC7154553; doi:10.1111/bjh.16296)
Supplement: Supplementary file 1 — Data S1. Supplementary methods. Table SI. Baseline patient characteristics. Table SII. Number of patients who received less than 90% of the intended dose for each of the drugs in each cycle, by treatment group. Table SIII. Schedule of investigations and follow‐up. Table SIV. Radiotherapy outlining protocol variations (one major variation patient displayed two types of variation). Table SV. FDG‐PET response categories. Table SVI. Worst grade of neurotoxicity during the trial. Table SVII. Non‐neuropathy toxicities: worst toxicity grade at any point in time of the trial (excluding neurotoxicity). Table SVIII. Worst grade of radiotherapy toxicity reported six months after completing radiotherapy. Fig S1. Consort diagram. Fig S2. Disease‐free survival. Fig S3. Sensory and motor quality‐of‐life scales. There was a significant deterioration in both scales between pretreatment and postchemotherapy. At 12 months post‐treatment, sensory scale was not significantly different to baseline but motor scale remained 2.6 points worse than at baseline. [file BJH-189-128-s001.docx]

# Supplementary data

## Supplementary introduction:

**Summary of evolution of treatment in the paediatric trials:**

In the GPOH-HD95 trial, all patients received 2 cycles of induction chemotherapy with OEPA (vincristine, etoposide, prednisolone, and doxorubicin) or OPPA (vincristine, procarbazine, prednisolone, and doxorubicin) and patients with intermediate and advanced stage disease received an additional 2 or 4 cycles of COPP (cyclophosphamide, vincristine, procarbazine, and prednisolone), respectively. Radiotherapy was omitted for patients in CR by conventional radiology criteria after chemotherapy and, although the results showed a promising 5-year EFS and overall survival (OS) of 89% and 97%, respectively, more relapses were identified in intermediate and advanced stage patients who did not receive radiotherapy suggesting that these patients were undertreated. (Dorffel, Ruhl et al. 2013). In the GPOH-HD-2002 trial, radiotherapy was omitted only for early stage patients in CR after 2 cycles of chemotherapy. The fertility sparing regimen COPDAC (in which dacarbazine replaced procarbazine) was used instead of COPP in males, giving 5 year EFS and OS of 92.0% and 99.5% in treatment group (TG) 1 and 87.7% and 96.2% for TG2 and TG3 combined, respectively (Mauz-Korholz *et al.* 2010).

**Further differences between paediatric and adult treatment regimens:**

The paediatric regimens have a higher initial intensity but lower cumulative dose of anthracycline than ABVD; 2 cycles of OEPA over 8 weeks contains the equivalent anthracycline dose as 3 cycles of ABVD over 12 weeks. Despite the initial high intensity of treatment, patients with advanced stage disease receive a lower total dose of anthracycline with 2 cycles of OEPA followed by COPP than if treated with 6 cycles of ABVD (160mg/m^2^ compared to 300mg/m^2^). Corticosteroids are used in high doses in the paediatric regimens with 15 days of prednisolone in each cycle whereas ABVD does not include steroids.

## Supplementary methods:

### Inclusion and exclusion criteria:

**Inclusion Criteria**

i. Biopsy proven de-novo classical Hodgkin lymphoma

ii. No previous chemotherapy or radiotherapy

iii. Age 18-30 years

iv. Any stage

v. Able to give informed consent

vi. Agreement to take adequate precautions to prevent conception during chemo-/radiotherapy and for up to one year afterwards

**Exclusion criteria**

i. Nodular Lymphocyte Predominant Hodgkin lymphoma

ii. Previous chemotherapy or radiotherapy

iii. Known or suspected HIV infection

iv. Pre-existing neurological disorder

v. Serious co-morbidity which may prevent administration of study treatment

vi. Prior organ transplant

vii. Previous malignancy

viii. Pregnancy or lactation

ix. Creatinine >1.5 upper limit of normal (ULN) not due to the lymphoma

x. ALT/AST/Bilirubin >2.5 ULN not due to the lymphoma. Patients with Bilirubin levels of >2.5 ULN due to Gilberts syndrome will be included.

### Staging and response assessment

All patients were staged with a contrast-enhanced CT scan of the neck, chest, abdomen and pelvis and FDG-PET. Bone marrow biopsy was performed if the stage was greater than 2A. Stage was determined according to the Cotswold revision of the Ann Arbor staging system taking into account the results of the FDG-PET scan (Lister, Crowther et al. 1989) . Areas of abnormal FDG uptake identified on the FDG-PET scan were only considered as involved sites of disease if there was evidence from conventional imaging to support the FDG-PET findings.

### PET Protocol

Patients fasted for 6 hours prior to scanning and drank 2-3 glasses of water to ensure adequate hydration. 4.5MBq/Kg of FDG was injected with uptake time of 90 minutes prior to scan acquisition. Whole body (vertex to upper thighs) PET data was acquired and reconstructed using OSEM. A low dose CT without IV contrast was acquired with the same coverage as the PET scan. Local site protocol determined use of PET in 2D or 3D mode and CT imaging parameters. Baseline and subsequent scans were acquired on the same scanner for each patient using the same patient preparation and imaging parameters.

Baseline scans were submitted for central review within 4 weeks of trial entry for confirmation of staging and final allocation of treatment group. Scans performed at end-of-chemotherapy were centrally reviewed by two independent reviewers to confirm the response to treatment and to determine radiotherapy volumes.

Central review of staging differed from local review in 2 cases and in both cases the patients were allocated to TG3 whereas local review had indicated that they should be in TG1 or TG2.

### Response assessment according to GPOH criteria

The protocol was designed before the introduction of international standardised response criteria incorporating PET results. Response assessment was performed according to the GPOH-HD definitions in use at the time the protocol was devised in which CR was attained if all sites of disease decreased in size by >95% and any residual masses measured ≤2ml in volume, non-measurable sites of disease were undetectable, disease symptoms had abated, and FDG-PET was negative. Good partial remission (GPR) was achieved when all sites of measurable disease had reduced by >75% but the criteria for CR were not met. Partial remission (PR) was achieved when all sites of disease had reduced by 50-75% from baseline (Mauz-Korholz *et al.* 2010). FDG-PET scans were defined as negative if there was complete resolution of all sites of uptake identified at baseline and as positive if abnormal FDG uptake was seen (Table S5).

### Treatment details:

OEPA consisted of vincristine 1.5mg/m^2^ IV (capped at a maximum of 2mg) on days 1, 8, and 15, etoposide 125mg/m^2^ IV on days 1 to 5, prednisolone 60mg/m^2^ orally on days 1 to 15, and doxorubicin 40mg/m^2^ IV on days 1 and 15. COPP consisted of cyclophosphamide 500mg/m^2^ IV on days 1 and 8, vincristine 1.5mg/m^2^ IV (capped at a maximum of 2mg) on days 1 and 8, procarbazine 100mg/m^2^ orally on days 1 to 15, and prednisolone 40mg/m^2^ orally on days 1 to 15. Cycle duration of both OEPA and COPP was 28 days.

Supportive care was recommended with anti-*pneumocystis jiroveci*, and anti-viral prophylaxis during treatment. Granulocyte colony stimulating factor (GCSF) was recommended if neutropenia was experienced to prevent treatment delays.

### Radiotherapy delivery:

All radiotherapy patients were planned using 3D-CRT. An involved site Clinical Target Volume (CTV) was delineated on CT following the Involved Site Radiotherapy (ISRT) principles described in the National Cancer Research Institute Lymphoma Radiotherapy Group Guidelines (Hoskin *et al.* 2013). Patients in TG2 or TG3 in CR received 20Gy ISRT to all initially involved sites of disease regardless of the FDG-PET scan result at end-of-chemotherapy. Patients in good PR (GPR) with >75% reduction of all sites of disease (regardless of PET findings) or PR (50-75% reduction in sites of disease volume) with a negative PET scan received 20Gy ISRT plus a 10Gy boost to residual masses measuring >50ml. Residual disease was outlined as the Gross Tumour Volume and a boost CTV defined as an expansion of this, by 15mm in all directions, constrained to anatomical boundaries. Patients in PR with PET-positive sites of disease received 30Gy ISRT to all initial sites of disease with no additional boost to residual sites of disease (Figure 1). Radiotherapy was delivered in 2Gy fractions, 5 days a week, and was subject to a central quality assurance assessment.

### Statistical considerations:

Sample size calculation was based on a Fleming single stage design. With a 90% power, one-sided 10% significance level and assuming the true neurotoxicity rate in the experimental treatment is ≤4%, 45 patients would be required to exclude a neurotoxicity rate of >15%. Also, with one sided significance level of 10% and 90% power, 45 patients would be sufficient to exclude a response rate of <80%, assuming that the true response is ≥93%.

For adverse events and neurotoxicity, the worst grade for each patient is presented. ORR is reported with respective 2-sided 80% CI. Time-to-event endpoints were DFS (defined as the time from documentation of CR to relapse or death from any cause) and OS (defined as the time from registration to death from any cause), and standard survival analysis was implemented.

QoL values for each of the scales obtained at each timepoint were compared with baseline values using paired t-tests. 99% CI were used and statistical significance was only assumed if P<0.01 for all QoL data to account for multiple testing.

## Supplementary tables:

|  | | |
| --- | --- | --- |
| Characteristic | No of patients | % |
|  |  |  |
| **All patients** | 47 | 100 |
|  |  |  |
| **Sex** |  |  |
| Female | 22 | 47 |
| Male | 25 | 53 |
| **Stage** |  |  |
| II | 28 | 60 |
| III | 6 | 13 |
| IV | 13 | 28 |
| **B symptoms** |  |  |
| Absent | 25 | 53 |
| Present | 22 | 47 |
| **ECOG performance status** |  |  |
| 0 | 41 | 87 |
| 1 | 5 | 11 |
| 2 | 1 | 2 |
| **Extranodal involvement** |  |  |
| Yes (E lesion) | 7 | 15 |
| Yes (Stage IV) | 6 | 13 |
| No | 32 | 68 |
| Not reported | 2 | 4 |
| **Treatment group** |  |  |
| TG1 (early stages) | 16 | 36 |
| TG2 (intermediate stages) | 11 | 24 |
| TG3 (advanced stages) | 18 | 40 |
|  |  |  |

Supplementary Table 1 Baseline patient characteristics

| Treatment group | Cycles | Chemotherapy drugs | | | |
| --- | --- | --- | --- | --- | --- |
| TG1  (N=16) |  | Vincristine or Vinblastine** | Etoposide | Prednisolone* | Doxorubicin |
|  | OEPA 1 | 2 | 0 | 2 | 0 |
|  | OEPA 2 | 0 | 0 | 2 | 0 |
| TG2  (N=11) |  | Vincristine or Vinblastine | Etoposide | Prednisolone | Doxorubicin |
|  | OEPA 1 | 0 | 0 | 0 | 0 |
|  | OEPA 2 | 0 | 0 | 0 | 0 |
|  |  | Vincristine or Vinblastine | Cyclophosphamide | Prednisolone | Procarbazine |
|  | COPP 1 | 0 | 0 | 0 | 0 |
|  | COPP 2 | 1 | 0 | 1 | 0 |
| TG3  (N=18) |  | Vincristine or Vinblastine | Etoposide | Prednisolone | Doxorubicin |
|  | OEPA 1 | 1 | 0 | 2 | 0 |
|  | OEPA 2 | 1 | 0 | 1 | 1 |
|  |  | Vincristine or Vinblastine | Cyclophosphamide | Prednisolone | Procarbazine |
|  | COPP 1 | 1 | 0 | 0 | 0 |
|  | COPP 2 | 3 | 0 | 1 | 1 |
|  | COPP 3 | 4 | 0 | 0 | 0 |
|  | COPP 4 | 4 | 0 | 0 | 0 |

* 5 patients in OEPA cycle 1 received less than 90% of the intended dose of the prednisolone. One of those patients was the patient who withdrew in first cycle of OEPA and was not classified into a treatment group.

** Vinblastine was administered in place of vincristine in 3 of 45 (7%) patients receiving OEPA cycle 2, 5 of 29 (17%) patients receiving COPP cycles 1 and 2, and 3 of 18 (17%) patients receiving COPP cycles 3 and 4.

Supplementary table 2 Number of patients who received less than 90% of the intended dose for each of the drugs in each cycle by treatment group

|  | **Baseline** | **Prior to each cycle of chemotherapy** | **After 2 x OEPA** | **After final cycle of chemotherapy** | **After all treatment** | **3 months after completion of all treatment** | **6 months after completion of all treatment** | **9 months after completion of all treatment** | **12 months after completion of all treatment** | **Follow-up^b^** |
| --- | --- | --- | --- | --- | --- | --- | --- | --- | --- | --- |
| **Toxicity** | X | X |  | X | X | X | X | X | X | X |
| **QOL** | X | X | X | X | X | X | X | X | X | X |
| **CT/MRI scan** | X |  | X | X |  | X |  |  |  |  |
| **FDG-PET** | X |  | X ^a^ | X |  |  |  |  |  |  |

Supplementary Table 3 Schedule of investigations and follow up.

^a^ FDG-PET required after 2 cycles of OEPA in TG1 only

^b^ Follow-up every 4 months for years 2 and 3, every 6 months in years 4 and 5 and annually thereafter

| ***Minor variations*** | ***No. of Patients*** | ***Major variations*** | ***No. of Patients*** |
| --- | --- | --- | --- |
| SUP and/or INF margin variation <2.0 cm | 3 | SUP and/or INF margin variation ≥2.0 cm | 3 |
| Liver disease should not be treated | 1 | Skeletal sites uninvolved in CT & MR should not be treated | 1 |
| Splenic hilum inclusion | 1 | Nodal region missed | 1 |

Supplementary Table 4 Radiotherapy outlining protocol variations (one major variation patient displayed 2 types of variation)

| **FDG-PET Result** | | **Definition** |
| --- | --- | --- |
| **Negative (1)** | | Complete disappearance of all abnormal uptake, highest residual uptake in tumour site less than or equal to mediastinal background |
| **Positive (2)** |  |  |
|  | **Partial Response** | Reduction in abnormal uptake but residual tumour activity greater than mediastinal background |
|  | **Stable** | No significant change compared to baseline |
|  | **Progression** | Increase in level of abnormal uptake or appearance of new sites |

Supplementary Table 5 FDG-PET Response Categories

| Neurotoxicity | Ever Worst Grade (CTCAE grade criteria*)  N (%**) | | | | Total |
| --- | --- | --- | --- | --- | --- |
|  | 1 | 2 | 3 | 4 |  |
| Motor | 9 (20%) | 6 (13%) | 2 (4%) | 0 (0%) | 17 (37%) |
| Sensory | 16 (35%) | 17 (37%) | 3 (7%) | 0 (0%) | 36 (78%) |
| Ileus/GI | 8 (17%) | 6 (13%) | 1 (2%) | 0 (0%) | 15 (33%) |
| Other not specified | 2 (4%) | 1 (2%) | 1 (2%) | 0 (0%) | 4 (9%) |
| Any neurotoxicity | 15 (33%) | 18 (39%) | 5 (11%) | 0 (0%) | 38 (83%) |
| * Patients who reported neurotoxicity related adverse events are taken into account in the table | | | | | |
| ** Percentages based on a total of 46 patients (47 patients were registered into the trial, one withdrew consent before receiving treatment) | | | | | |

Supplementary Table 6 Worst grade of neurotoxicity during the trial

| Toxicities  (CTCAE grade criteria) | Grades | |
| --- | --- | --- |
|  | 3 | 4 |
|  | N (%) | N (%) |
| **HAEMATOLOGICAL** |  |  |
| Haemoglobin decreased | 1 (2%) | 0 (0%) |
| Neutrophil count decreased | 9 (20%) | 29 (63%) |
| Platelet count decreased | 5 (11%) | 3 (7%) |
| White blood cell decreased | 7 (15%) | 0 (0%) |
| **NON-HAEMATOLOGICAL** |  |  |
| **Constitutional symptoms** |  |  |
| Fatigue | 2 (4%) | 0 (0%) |
| Fever | 2 (4%) | 0 (0%) |
| **Gastrointestinal** |  |  |
| Nausea | 3 (7%) | 0 (0%) |
| Vomiting | 3 (7%) | 0 (0%) |
| Diarrhoea | 2 (4%) | 0 (0%) |
| Mucositis/stomatitis | 6 (13%) | 0 (0%) |
| Ascites | 1 (2%) | 0 (0%) |
| **Pain** |  |  |
| Gastrointestinal | 4 (9%) | 0 (0%) |
| Musculoskeletal | 2 (4%) | 1 (2%) |
| NOS | 1 (2%) | 0 (0%) |
| Other * | 1 (2%) | 0 (0%) |
| **Infection** |  |  |
| Febrile neutropenia | 9 (20%) | 1 (2%) |
| **Vascular** |  |  |
| Thrombosis/embolism | 2 (4%) | 0 (0%) |
| **Cardiac** |  |  |
| Hypotension | 1 (2%) | 1 (2%) |
| **Respiratory** |  |  |
| Pleural effusion | 1 (2%) | 0 (0%) |
| **Osteonecrosis** |  |  |
| Osteonecrosis ^**^ | 2 (4%) | 0 (0%) |
| ^*^ Patient with grade 3 pain had pelvic pain due to osteonecrosis | | |
| ^**^ One other patient had grade 2 of osteonecrosis | | |

Supplementary Table 7 Non-neuropathy toxicities: worst toxicity grade at any point in time of the trial (excluding neurotoxicity).

| Worst grade reported of radiotherapy toxicity (RTGO criteria) | Patients who received radiotherapy (N=41) | | | | | |  |
| --- | --- | --- | --- | --- | --- | --- | --- |
|  | Mild | | Moderate | | Severe | | Total (%) |
|  | N | % | N | % | N | % |  |
| Skin problems § | 3 | 7 | 2 | 5 | 0 | 0 | 5 (12%) |
| Mucositis ∫ | 8 | 20 | 2 | 5 | 2 | 5 | 12 (29%) |
| Gastrointestinal ‡ | 5 | 12 | 2 | 5 | 0 | 0 | 7 (17%) |
| Other acute toxicity* | 10 | 24 | 2 | 5 | 2 | 5 | 14 (34%) |
| Any radiotherapy toxicity | 16 | 39 | 4 | 10 | 3 | 7 | 23 (56%) |
| § Skin problems: 2 patients with moderate Erythema | | | | | | | |
| ∫ Mucositis: 1 patient with moderate oral mucositis, 1 patient with moderate mouth mucositis, 1 patient with severe throat and mouth mucositis, 1 patient with severe mouth and oesophagus mucositis | | | | | | | |
| ‡ Gastrointestinal: 1 patient with moderate Nausea, 1 patient with moderate Diarrhoea | | | | | | | |
| * Other acute toxicity: 1 patient with moderate Anorexia, 1 patient with moderate abnormal taste perception; 1 patient with severe Frontal headache, 1 patient with severe dysphagia | | | | | | | |

Supplementary table 8 Worst grade of radiotherapy toxicity reported 6 months after completing radiotherapy

## Supplementary figures:


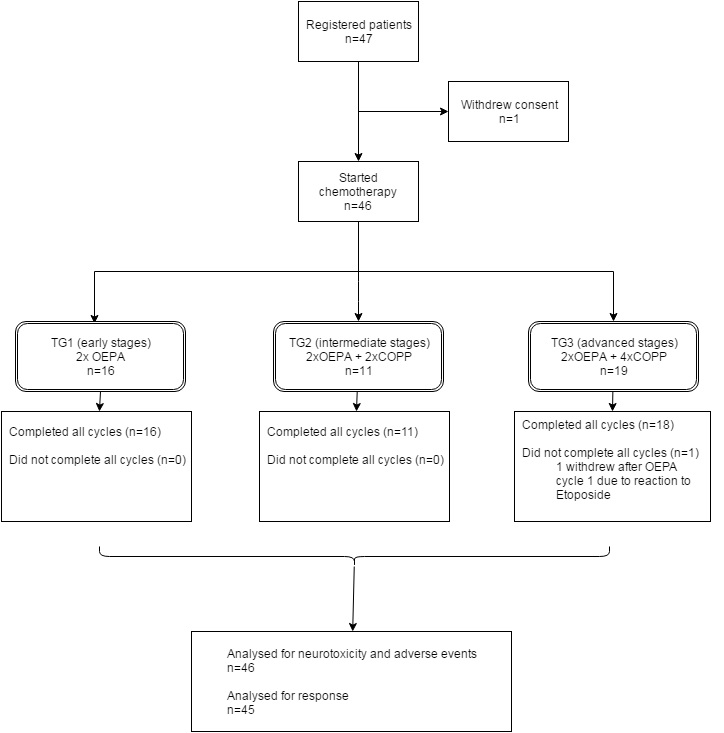


Supplementary Figure 1: Consort diagram

Supplementary Figure 2 Disease free survival

Supplementary Figure 3 Sensory and Motor quality of life scales. There was a significant deterioration in both scales between pre-treatment and post-chemotherapy. At 12 months post treatment, sensory scale was not significantly different to baseline but motor scale remained 2.6 points worse than at baseline.

## Supplementary References:

Lister, T.A., Crowther, D., Sutcliffe, S.B., Glatstein, E., Canellos, G.P., Young, R.C., Rosenberg, S.A., Coltman, C.A. & Tubiana, M. (1989) Report of a committee convened to discuss the evaluation and staging of patients with Hodgkin’s disease: Cotswolds meeting. *Journal of Clinical Oncology*, **7,** 1630–1636.
